# Supplementary material for: The Sweetpotato Voltage-Gated K+ Channel β Subunit, KIbB1, Positively Regulates Low-K+ and High-Salinity Tolerance by Maintaining Ion Homeostasis
Source: Genes (Basel). 2022 Jun 20;13(6):1100. doi: 10.3390/genes13061100 (PMC9222298; doi:10.3390/genes13061100)
Supplement: Supplementary file 1 [file genes-13-01100-s001.zip › Supplementary materials.pdf]

Table S1. Primers used in this study

Primer name	Primer scequence（5'→3'）
IbActin-F:	AGCAGCATGAAGATTAAGGTTGTAGCAC
IbActin-R:	TGGAAAATTAGAAGCACTTCCTGTGAAC
KIbB1-qPCR-F:	ACCCGATAGTAGGTTTGCAC
KIbB1-qPCR-R:	AATCTGGGCGCTTTGG
KIbB1-SacI-F:	CGAGCTCATGGATTATGTGGACTTGATCTACT
KIbB1-XbaI-R:	GCTCTAGAGCGATAAGAATCTGGGCGC
KIbB1-T-F:	GGTCGCGGAGGCTATGGATGC
KIbB1-T-R:	GCTTCTGCGGGCGATTTGTGT
Atactin-F	GCACCCTGTTCTTCTTACCGA
Atactin-R	AGTAAGGTCACGTCCAGCAAGG
AtCAT-F	GCAACTACCCCGAGTGGAAA
AtCAT-R	TGTTCAGAACCAAGCGACCA
AtGPX8-F	ATGGCGACGAAGGAACCAG
AtGPX8-R	ATCGCCGAAGATTCCCCATTT
AtP5CS-F	ATGATCTTATTTATGTTCTGC
AtP5CS-R	CACTATCTTCCGTCACTAT
AtP5CR-F	AGTTTAGCTTCACAGACCGTTC
AtP5CR-R	GCTCTGTGAGAGCTCGCGGCTTC
AtDHAR-F	ATGGTCCTTTTATCGCCGGG
AtDHAR-R	GCCCATCCAGAGATCACACA
AtAPX-F	CTCTGGGACGATGCCACAAG
AtAPX-R	CTCGACCAAAGGACGGAAAA
AtPOD-F	TCCGGGAGCCACACCATTGG
AtPOD-R	TGGTCGGAATTCAACAG
AtSOD-F	ATGAGAAGTTCTATGAAGAG
AtSOD-R	GTCTTTATGTAATCTGGT

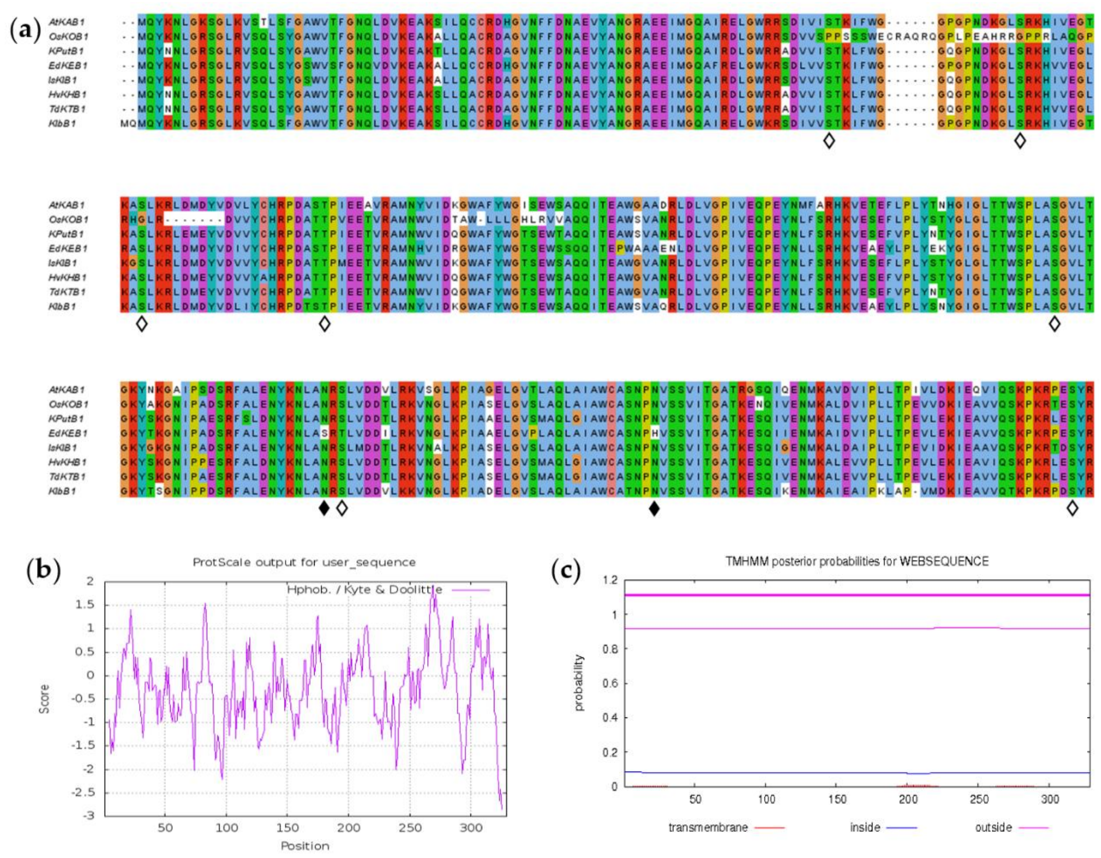

Figure S1. Amino acid sequence analysis of KIbB1. (**a**) Multiple amino acid sequence alignment of KIbB1 with homologs from NCBI. (**b**) The polarity prediction of KIbB1. The positive value indicated hydrophobicity and the negative value indicated hydrophilicity. (**c**) Transmembrane domain prediction of KIbB1.

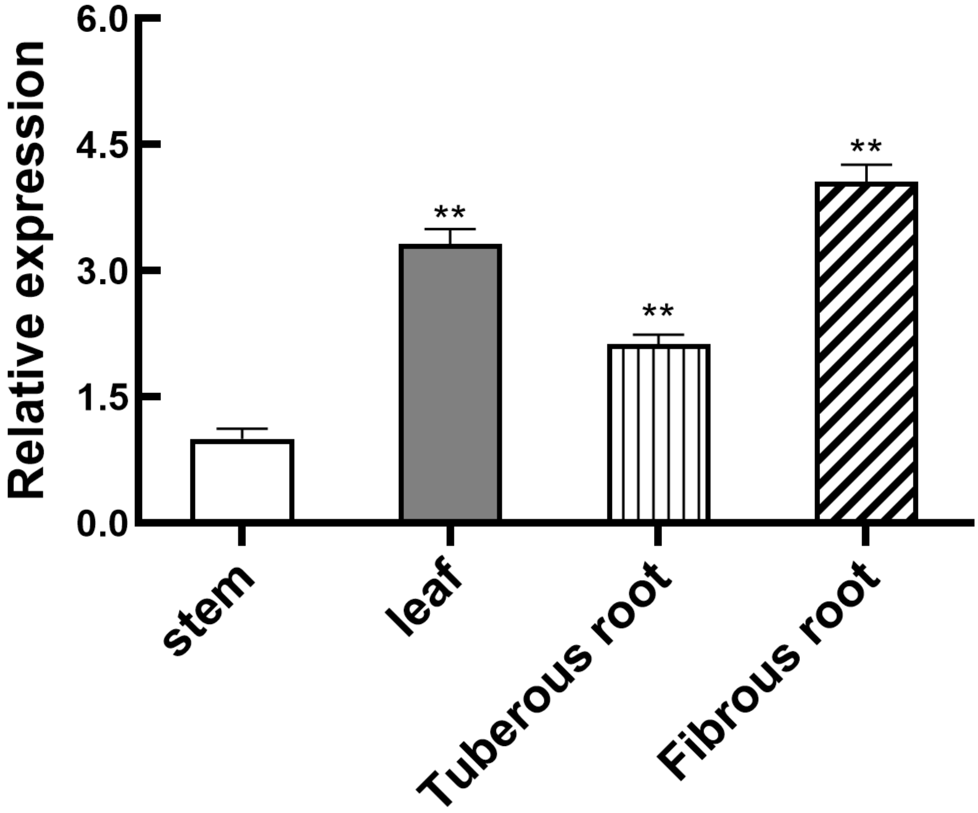

Figure. S2 Expression analysis of *KIbB1* in different tissues. Data are presented as means ± SE (n = 3). ** indicated a significant difference at $p < 0.01$.

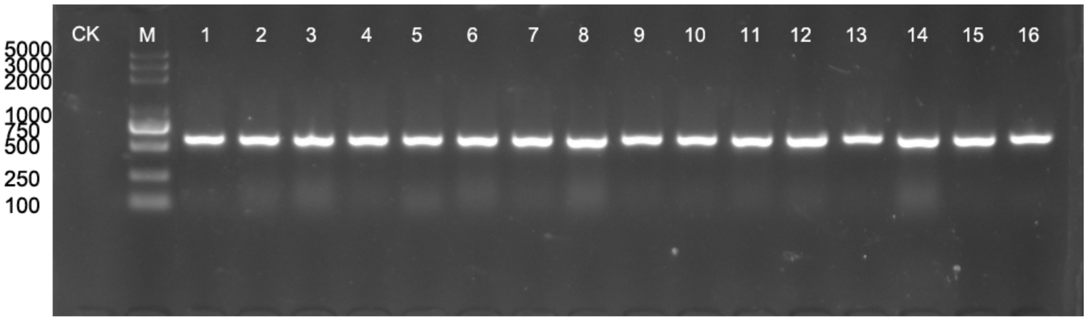

Figure S3. PCR identification of *KIbB1* transgenic plants. M, DNA marker; CK, negative control; 1-16, candidate transgenic plants.
